# Comment on “Diagnostic Potential of Macromolecular Proton Fraction Mapping Combined with Quantitative Susceptibility Mapping as a Subcortical Biomarker for Parkinson’s Disease” by Fujiwara et al.

**DOI:** 10.2463/mrms.lte.2025-0176

**Published:** 2026-02-04

**Authors:** Rohma Zubairi

**Affiliations:** Jinnah Sindh Medical University, Karachi, Pakistan

**Keywords:** biomarkers, Parkinson’s disease, quantitative magnetic resonance imaging

## To the Editor,

I read with great interest the recent article by Fujiwara et al.^1^ describing the integration of macromolecular proton fraction (MPF) mapping with quantitative susceptibility mapping (QSM) as a composite subcortical biomarker for Parkinson’s disease (PD). The authors should be commended for exploring this innovative multiparametric approach. However, we wish to highlight several aspects that merit further consideration before these findings can be generalized.

First, the study cohort (20 PD patients and 9 controls) is relatively small, and no power calculation was presented. Given the high interindividual variability in both MPF and QSM metrics, the limited sample size may overestimate diagnostic separation. Larger, multicenter cohorts would be necessary to validate reproducibility and normative thresholds.

Second, while MPF and QSM individually capture complementary tissue microstructure and iron load,^2^^,^^3^ the physiological interpretation of their combined alterations remains ambiguous. The observed correlations could reflect non-specific iron and myelin interactions rather than disease-specific pathology. Clarifying the biological basis, possibly through phantom validation or histopathologic correlation, would strengthen the mechanistic inference.^4^

Third, the study lacks discussion on cross-vendor and field-strength harmonization. Both MPF and QSM are highly sensitive to sequence parameters and reconstruction pipelines, so without inter-scanner reproducibility testing, clinical translation remains uncertain. Providing raw data ranges or coefficients of variation would enhance transparency and comparability.^2^

Finally, it would be valuable to contextualize these results against existing PD imaging biomarkers such as neuromelanin-sensitive MRI and diffusion tensor metrics.^5^ Demonstrating additive diagnostic value beyond established techniques could reinforce the clinical impact of this combined approach.

I appreciate the authors’ contribution and hope these suggestions encourage further standardization and validation efforts to advance quantitative MRI biomarkers in Parkinson’s disease.

Sincerely,

Rohma ZubairiJinnah Sindh Medical University zubairirohma@gmail.com

## Reply to “Letter to the Editor”

Yasuhiro Fujiwara^1^^*^, Nobutaka Sakae^2^, Hiroyuki Kumazoe^3^, Kouta Miyamoto^3^, Yoshinori Hirakawa^3^, Hirohito Kan^4^, Kosuke Yamashita^1^, and Mika Kitajima^5^

^1^Department of Medical Imaging Technology, Faculty of Life Sciences, Kumamoto University, Kumamoto, Kumamoto, Japan

^2^Department of Neurology, National Hospital Organization Omuta National Hospital, Omuta, Fukuoka, Japan

^3^Department of Radiology, National Hospital Organization Omuta National Hospital, Omuta, Fukuoka, Japan

^4^Department of Integrated Health Sciences, Nagoya University Graduate School of Medicine, Nagoya, Aichi, Japan

^5^Department of Diagnostic Imaging Technology, Faculty of Life Sciences, Kumamoto University, Kumamoto, Kumamoto, Japan

*Corresponding author: yfuji@kumamoto-u.ac.jp

## Dear Editor,

We greatly appreciate the insightful comments from Dr. Zubairi regarding our recently published article, “Diagnostic Potential of Macromolecular Proton Fraction Mapping Combined with Quantitative Susceptibility Mapping as a Subcortical Biomarker for Parkinson’s Disease,” in Magnetic Resonance in Medical Sciences.^1^

We acknowledge that the small sample size represents a significant limitation of our exploratory study. As noted in the Discussion section, this study was designed as a proof of concept to identify statistically significant differences in macromolecular proton fraction (MPF) and quantitative susceptibility mapping (QSM) measurements in Parkinson’s disease (PD). Although the observed group differences, reflected by the observed *P*-values and false discovery rate (FDR)–adjusted *P*-values, suggest that MPF and QSM are sensitive to PD-related changes, validation in larger, adequately powered cohorts are essential for establishing robust normative thresholds and ensuring reproducibility. Such studies are an important next step toward the clinical translation of quantitative imaging biomarkers.

We appreciate Dr. Zubairi’s thoughtful commentary on the physiological interpretation of the concurrent MPF and QSM changes. MPF reflects the macromolecular and myelin-related content, whereas QSM reflects iron deposition and other magnetic sources. The co-occurrence of these alterations may indicate convergent microstructural changes. However, we fully agree that MPF and QSM alone cannot determine the underlying biological mechanisms, and the causal relationships among these changes remain uncertain. Direct histopathological validation was not feasible in the current cohort. Accordingly, we interpreted our findings conservatively, in the context of established neuropathological literature. Further refinement may be aided by examining how MPF–QSM findings relate to other noninvasive quantitative MRI measures, although such analyses cannot substitute for pathological confirmation.

We appreciate the emphasis on reproducibility across different scanning platforms. All data were acquired on a single 3.0T MRI scanner using standardized acquisition and reconstruction protocols to minimize technical variability. Although cross-vendor and multisite harmonization were beyond the scope of this initial single-center study, we fully recognize their importance for eventual clinical translation. We hope that our preliminary findings will encourage broader collaborative efforts within the research community.

Finally, we agree that positioning the MPF–QSM within the broader landscape of PD imaging biomarkers is important. Our study was designed to assess the feasibility and diagnostic potential of combining MPF and QSM rather than performing head-to-head comparisons with other modalities. Nevertheless, evaluating whether MPF–QSM provides complementary or incremental diagnostic value over established biomarkers such as neuromelanin-sensitive MRI and diffusion tensor imaging is important to determine its optimal role within a multimodal imaging framework. We consider these comparative investigations a valuable direction for future research.

We thank Dr. Zubairi for these constructive suggestions, which highlighted key considerations for advancing quantitative neuroimaging in PD. Multiparametric MRI approaches, including a combination of MPF and QSM, hold considerable promise for the development of objective imaging biomarkers. The methodological challenges identified, including validation in larger cohorts, harmonization across platforms, and comparison with established techniques, represent essential steps toward clinical translation that warrant continued attention from the research community.

Sincerely,

Yasuhiro Fujiwara, Nobutaka Sakae, Hiroyuki Kumazoe, Kouta Miyamoto, Yoshinori Hirakawa, Hirohito Kan, Kousuke Yamashita, and Mika Kitajima.
